# Structural studies of the N-terminal fragments of the WW domain: Insights into co-translational folding of a beta-sheet protein

**DOI:** 10.1038/srep34654

**Published:** 2016-10-04

**Authors:** Yuya Hanazono, Kazuki Takeda, Kunio Miki

**Affiliations:** 1 Department of Chemistry, Graduate School of Science, Kyoto University, Sakyo-ku, Kyoto, 606-8502, Japan

## Abstract

Nascent proteins fold co-translationally because the folding speed and folding pathways are limited by the rate of ribosome biosynthesis in the living cell. In addition, though full-length proteins can fold all their residues during the folding process, nascent proteins initially fold only with the N-terminal residues. However, the transient structure and the co-translational folding pathway are not well understood. Here we report the atomic structures of a series of N-terminal fragments of the WW domain with increasing amino acid length. Unexpectedly, the structures indicate that the intermediate-length fragments take helical conformations even though the full-length protein has no helical regions. The circular dichroism spectra and theoretical calculations also support the crystallographic results. This suggests that the short-range interactions are more decisive in the structure formation than the long-range interactions for short nascent proteins. In the course of the peptide extension, the helical structure change to the structure mediated by the long-range interactions at a particular polypeptide length. Our results will provide unique information for elucidating the nature of co-translational folding.

Full-length proteins can fold into thermodynamically stable structures at an exceptionally fast rate, as shown by *in vitro* experiments. For example, several proteins consisting of a few tens of amino acid residues have been shown to fold in a few microseconds^1^,^2^. On the other hand, the codon translation ratio is 20–30 amino acid residues per second in *E. coli* or 2–4 amino acid residues per second in a typical eukaryote^3^. Therefore, co-translational folding of nascent proteins can follow different pathways from the full-length protein folding^4^,^5^,^6^,^7^,^8^. In order to better understand co-translational folding, it will be of considerable importance to image the transient structures. However, it is difficult to identify the optimal *in vivo* conditions of the transient structures due to the dynamic nature of co-translational folding, which is influenced by the ribosomal surface^9^,^10^, molecular chaperones^11^,^12^, and crowding conditions^13^. Therefore, there have been only a small number of studies on co-translational folding, and these have tended to use simplified models with nuclear magnetic resonance^6^, fluorescence resonance energy transfer^7^, and computational methods^14^. Though it has been reported that nascent proteins on the ribosomes have a native-like structure^6^, the atomic-level details of co-translational folding are not yet clear.

In order to reveal the atomic-level details of co-translational folding, we determined the structures of a series of nascent model peptide with increasing amino acid length (Fig. 1a). For this study, we chose the human Pin1 Trp-Trp domain (WW domain), which consists of 35 residues, as our model peptide. The topology of the WW domain composed of three strands and two beta-hairpins, which form an antiparallel beta-sheet^15^. The WW domain folds independently and the folding mechanisms of the wild type and variants have been intensively investigated with nuclear magnetic resonance, spectroscopic and computational simulation methods^16^,^17^,^18^,^19^. In this study, we prepared WWX, where the X-residue N-terminal WW domain fragments is denoted using the format WWX (Fig. 1b). For example, the 10-residue N-terminal fragment is represented as WW10. In addition, we prepared WWX fused with maltose-binding protein at their C-termini (WWX-MBP) for crystallization.

## Results

### Crystal structure of the WW domain fragments

**Figure 2:**
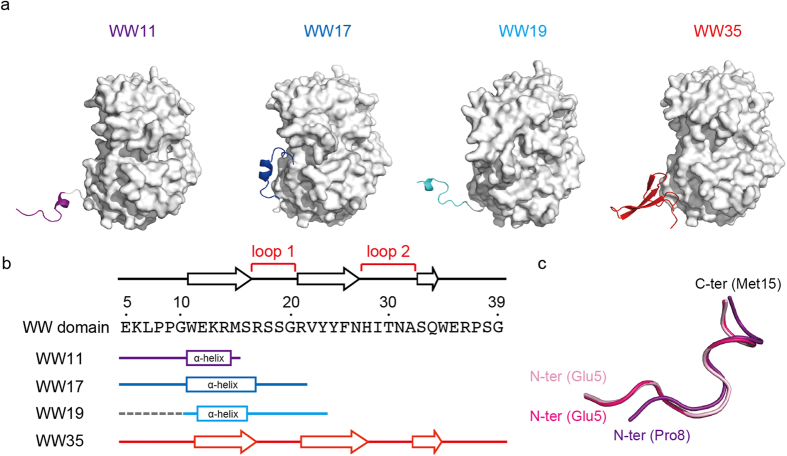
Crystal structures of the WW domain fused with MBP. (**a**) Overall structure of the WW domain fused with MBP. The regions of the WW domain and linker are shown as ribbon models and the regions of MBP are shown as surface models. (**b**) Secondary structure of the regions of the WW domain. The cylinders and arrows indicate alpha helices and beta strands, respectively. The disordered regions are shown as dashed lines. (**c**) Superimposition with three WW11. The (**a,b**) chains in the Form I crystal are colored magenta and pink, while the A chain in the Form II crystal is colored purple.

The structures of the intermediate-length (WW11-MBP, WW17-MBP, WW19-MBP) and full-length (WW35-MBP) WW domains, which are fused with MBP at their C-termini, were determined with X-ray crystallography (Supplementary Table 1). The regions of the WW domain are clearly visible in the electron density map for the WW domain (Supplementary Figs 1 and 2). WW11, WW17, and WW19 take an alpha-helical conformation (Fig. 2a,b). On the other hand, WW35 (full-length) has a three-stranded beta-sheet structure, as previously reported for the X-ray structure of the full-length WW domain^15^ (Fig. 2a,b). These structures can be superimposed with a root-mean-square deviation (RMSD) of 1.14 Å (Supplementary Fig. 3).

In order to assess the crystal packing effect, three independent structures of WW11 in two crystal forms were compared (Fig. 2c). Two chains of WW11 in Form I can be superimposed onto each other with an RMSD of 0.22 Å. In addition, the WW11 in Form II can be superimposed onto that in chain A of Form I with an RMSD of 1.65 Å. This indicates that there is little or no packing effect for the structures of the WW domain fragments.

**Figure 3:**
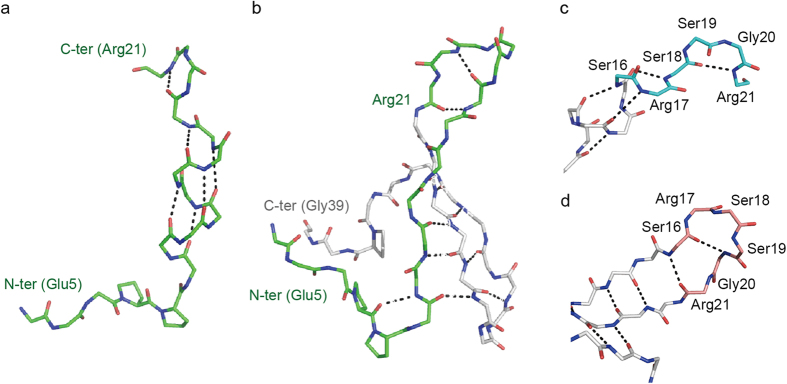
Conformations of the regions of the WW domain. (**a**) Backbone diagram of WW17 shown as a stick model. Hydrogen bonds are shown as black dashed lines. (**b**) Backbone diagram of WW35. Residues 5–21 are colored green and the others are colored gray. (**c**) Close-up view of the loop 1 region (residues Ser16–Arg21) of WW17. Residues 16–21 are colored cyan and the others are colored gray. (**d**) Close-up view of the loop 1 region (residues Ser16–Arg21) of WW35. Residues 16–21 are colored salmon pink and the others are colored gray.

The structures of WW17 and WW19 form helices in the same manner as WW11, in which the region of the residues 12–15 forms helical structure (Fig. 2a,b). The most prominent difference between WW17 and WW19 is the torsion angles between residues Ser16 and Arg17, however, they share essentially the same helical structure. Consequently, the hydrogen bond networks of the intermediate-length WWX are distinctly different from that of WW35 (Fig. 3a,b). Though WW17 harbors residues 17–20, which form hairpin loop 1 in WW35, their conformations are not identical to WW35 (Fig. 3c,d). In WW35, the loop arises through an interaction between the backbone amide N of Ser16 and the carbonyl O of Arg21. However, in WW17, this hydrogen bond is not formed (Fig. 3c). WW19 also forms no beta-sheets (Supplementary Fig. 4), even though there are four potential interacting pairs of atoms (O of Ser16 to N of Gly20, N of Ser16 to O of Arg21, O of Arg14 to N of Tyr23, and N of Arg14 to O of Tyr23) involved in the antiparallel beta-sheet in the WW35 (Fig. 3d). This result indicates that the hairpin loop 1 does not form the native-like conformation in the transient structure.

### Spectroscopic analysis of the WW domain fragments

We attempted the crystallizations of WW23-MBP, WW27-MBP, and WW31-MBP, but we were unable to obtain crystals for these proteins. In order to obtain structural information for various additional WWX fragments, we collected circular dichroism (CD) spectra (Supplementary Fig. 5a). In the presence of 30% 2,2,2-trifluoroethanol (TFE), which simulates the physiological conditions^20^,^21^, WW17 and WW19 contain about 25% helices and do not contain beta-strands (Supplementary Fig. 5b,c). WW23 and WW27 mainly contain beta-stranded conformations under this condition. WW31 and WW35 have beta-stranded conformations. The CD analysis results indicate that the helical structures are unique to intermediate-length WWX fragments. Additionally, in the case of WW35, the circular dichroism spectrum is not affected by the concentration of TFE. The conformation of WW35 is more tolerant to guanidine hydrochloride than that of WW31 based on the tryptophan fluorescence spectroscopy (Supplementary Fig. 6). Therefore, the folding of the nascent WW domain is eventually stabilized after translation of the full-length domain.

## Discussion

In this study, we found that the intermediate-length fragments of the WW domain form alpha-helical structures. In addition, the crystal structures of WW17 and WW19 revealed that the region of loop 1 is different from that of the wild type. These results show that the structures of the nascent proteins are determined by already synthesized polypeptide sequences.

The structures of the WW domain fragments were also examined with a *de novo* approach^22^ (Supplementary Fig. 7). The three-stranded beta sheet-conformation of WW35 is correctly reproduced by the method. The predicted structures of WW11, WW15 and WW35 are nearly identical to the crystal structures. Therefore, the helical conformations in the crystals are not artifacts. We also predicted the conformations of WW23, WW27 and WW31, whose crystals structures were not resolved, in the same manner (Supplementary Fig. 7). WW23 and WW27 form beta-hairpin structures, while WW31 forms two beta-hairpins between three-stranded beta-sheets in the same way as WW35. These predicted models are in good accord with the CD spectroscopic results.

**Figure 4:**
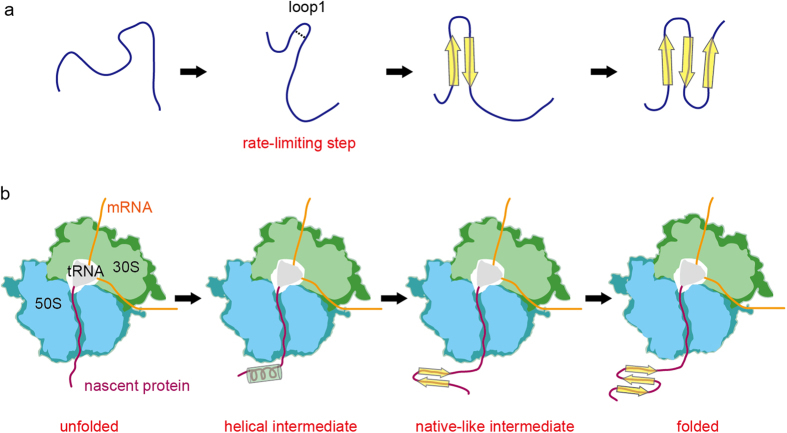
Schematic representations of the folding pathway. (**a**) Full-length folding pathway of the WW domain. Hydrogen bonds are shown as black dashed lines. (**b**) Co-translational folding pathway of the WW domain. The cylinders and arrows indicate alpha helices and beta strands, respectively.

In the case of full-length folding, it has been considered that the WW domain initially forms the first beta-hairpin (loop 1), which is the rate-limiting step^16^,^17^, and then folds into the beta-sheet structure, based on the results of mutational^18^ and computational^19^ analyses (Fig. 4a). These studies commonly suggest that the folding of the WW domain proceeds as a downhill process. On the other hand, our results suggest that the co-translational folding of the WW domain initially forms a helical-structure even though the sequence of loop 1 exists (Fig. 4b). When the intermediate-length WW domain becomes long enough to maintain a beta-sheet structure using beta 1 and beta 2, it is plausible that it forms one beta-hairpin structure.

The long-range interactions are of great importance for the stability of the globular proteins^23^. However, the structures of the intermediate-length fragments of the WW domain are significantly influenced by the short-range interactions in helical conformations according to the crystallographic, CD spectroscopic and structural prediction results. On the other hand, the residues 17–20 at the C-termini of WW17 and WW19, where hairpin loop 1 is formed in WW35, take more extended conformations, while the conformations are stabilized by a kind of the short-range interactions (Fig. 3d and Supplementary Fig. 4). It is plausible that the residues 17–20 are the origin of the transition from the short-range interaction to the long-range interaction because this region has a low propensity to take helical conformation. The helical structure mediated by the short-range interactions may change to that by the long-range interactions at a particular peptide length during the peptide extension.

The nascent proteins are synthesized on the ribosome and exit through the ribosomal tunnel. A previous theoretical analysis indicated that the newly synthesized polypeptides are predisposed toward the helical structure in the tunnel^24^. In fact, it has been revealed that some nascent polypeptides form helical or helical-like conformation in the ribosomal tunnel using single-particle cryo-electron microscopy^25^,^26^,^27^. Therefore, it is also possible that nascent proteins form the helical conformation inside of the ribosomal tunnel and stabilize the helical structure outside of the tunnel. Our results indicate that transient helices can be formed even in the beta proteins at the protein extension. The folding pathway via the helical conformation may be essential for many beta proteins.

This study shows the helical folding of N-terminal fragments of a beta protein at atomic resolution. Our results are expected to give rise to further experimental and theoretical studies on co-translational folding.

## Methods

### Preparation of proteins and peptides

The WW17, WW19, WW23, WW27, WW31 and WW35 peptides were synthesized by the Fmoc solid-phase method and purified to >95% by the CS Bio Co. The genes for expression of the WW domain fragment fused with MBP at its C-terminus were inserted into a pET22b vector (NdeI/HindIII site). The linker sequences, which were Gly-Ser-Gly for WW11 and WW19 and Gly-Ser-Gly-Met for WW17, were inserted between the WW domain fragment and MBP. The fragments of the MBP and WW domain were amplified from pKM596 vector^28^ (Addgene plasmid 8837) and artificial gene synthesis (Hokkaido System Science), respectively. These constructs were transformed into Rosetta2(DE3)pLysS and grown at 37 °C in LB medium containing 100 μg/mL ampicillin and 34 μg/mL chloramphenicol. The protein expressions were induced by addition of 1 mM IPTG at an optical density at 600 nm of 0.6 at 37 °C for 3 hours. After the cells were harvested, the pellet was resuspended in 50 mM Tris-HCl (pH 7.5) and 150 mM NaCl (Buffer A) and disrupted by sonication. The suspension of disrupted cells was centrifuged at 40000 × *g* for 30 minutes at 4 °C. The supernatant was applied to an MBPTrap column (GE Healthcare) equilibrated with Buffer A. The bounded proteins were eluted with Buffer A containing 10 mM maltose. Then the pooled samples were applied to a HiLoad 16/60 Superdex 200 column (GE Healthcare) equilibrated with Buffer A.

### Crystallographic analysis

The proteins of WWX-MBP were concentrated to 20 mg/mL in 10 mM Tris-HCl (pH 7.5), 150 mM NaCl and 10 mM maltose. All crystallization attempts were made using the vapor-diffusion method at 293 K. Crystals were obtained in a solution that was a 1:1 mixture of protein solution and reservoir solution. The reservoir conditions differed for the individual variants as follows: 1.8 M ammonium citrate was used for Form I of WW11-MBP, 2.2 M DL-malic acid for Form II of WW11-MBP, 2.1 M DL-malic acid for WW17-MBP, 1.6 M ammonium citrate for WW19-MBP, and 1.6 M ammonium citrate for WW35-MBP. The crystals were flash cooled in a cold stream of nitrogen after soaking in the cryoprotectant solutions consisting of reservoir solution with 20% (*w/v*) glycerol. The X-ray diffraction data were collected with synchrotron X-rays from BL41XU (1.0000 Å, 100 K), BL44XU (0.9000 Å, 100 K) of SPring-8 (Harima, Japan), or with Cu*K*α X-rays from an in-house rotating anode X-ray generator MicroMax-007 (1.5418 Å, 100 K) (Rigaku). Diffraction data sets were processed and scaled using the HKL2000 software package^29^. Form I of WW11-MBP is in orthorhombic crystal form and belongs to the space group *C*222_1_ with unit-cell parameters *a* = 97.5 Å, *b* = 126.1 Å, *c* = 173.5 Å. There are two molecules in an asymmetric unit. Form II of WW11-MBP is in tetragonal crystal form and belongs to the space group *P*4_1_2_1_2 with unit-cell parameters *a* = 115.6 Å, *b* = 115.6 Å, *c* = 55.6 Å. There is one molecule in an asymmetric unit. The crystals of WW17-MBP and WW19-MBP belong to space group *P*2_1_2_1_2_1_, with unit-cell parameters *a* = 51.2 Å, *b* = 73.0 Å, *c* = 100.8 Å and *a* = 48.3 Å, *b* = 57.7 Å, *c* = 124.2 Å, respectively. The crystals of WW17-MBP and WW19-MBP contain one molecule in an asymmetric unit. The crystals of WW35-MBP belong to space group *P*2_1_, with unit-cell parameters *a* = 84.5 Å, *b* = 120.4 Å, *c* = 110.7 Å, *β* = 97.8°. There are four molecules in the asymmetric unit. All structures were solved by the molecular replacement method using MBP^30^ (PDBID: 1ANF) as a search model with the program Phaser^31^. The phenix.autobuild program^32^ was employed for the autotracing. The output structures were manually improved with the Coot program^33^. The structures were refined using the phenix.refine^32^. The validity of the refined structures was checked with the program MolProbity^34^. The crystallographic and refinement statistics are listed in Supplementary Table 1. The superimpositions were performed with the program LSQKAB^35^. The secondary structure was assigned using the program DSSP^36^. All figures for the molecular models were prepared using the program PyMOL^37^.

### CD spectroscopy

The CD spectra of the WW17, WW19, WW23, WW27, WW31 and WW35 peptides were measured in a range from 190 to 250 nm using a J-805 CD spectropolarimeter (Jasco) with a 1 mm quartz cuvette. All samples (0.1 mg/mL) were dissolved in 5 mM potassium phosphate buffer (pH 7.5) in the presence of 0–50% TFE at intervals of 10%. The secondary structure contents were analyzed with the program JWSSE-408 (Jasco). The CD spectra of WW31 and WW35 have positive ellipticity at 230 nm, which is due to an abundance of aromatic residues^38^. This positive ellipticity around 230 nm becomes an obstrucle in the estimation of the secondary structure contents. Therefore, we predicted the secondary structures using a reference data set^39^ with the spectra of N-acetyl-L-phenylalanineamide, N-acetyl-L-tryptophanamide and N-acetyl-L-tyrosineamide in accordance with the protein sequence (Supplementary Fig. 8).

### Tryptophan fluorescence

The fluorescent spectra of WW31 and WW35 peptides were measured using F-2500 Fluorescence Spectrophotometer (Hitachi) with an excitation at 280 nm. All samples (500 nM) were dissolved in 0–5 M guanidine hydrochloride and 50 mM potassium phosphate buffer (pH 7.5). Each spectrum was the average of three individual measurements. The denaturation curves were fitted to a Boltzmann sigmoidal function.

### Conformational modeling

Conformational modeling of the WW domain fragments was performed using the program PEP-FOLD^22^ (web server http://bioserv.rpbs.univ-paris-diderot.fr/PEP-FOLD/). The structural construction and energy evaluation depend on the OPEP (optimized potential for efficient structure prediction) coarse-grained force field. The sequences of WW11, WW15, WW19, WW23, WW27, WW31, and WW35 were submitted to the PEP-FOLD server. The secondary structure was assigned using the program DSSP^36^.

## Additional Information

****Accession codes**:** Coordinates and structure factors have been deposited in the Protein Data Bank under the accession number 5BMY, 5B3W, 5B3X, 5B3Y, and 5B3Z.

**How to cite this article**: Hanazono, Y. *et al*. Structural studies of the N-terminal fragments of the WW domain: Insights into co-translational folding of a beta-sheet protein. *Sci. Rep.* **6**, 34654; doi: 10.1038/srep34654 (2016).

## Supplementary Material

Supplementary Information

## Figures and Tables

**Figure 1:**
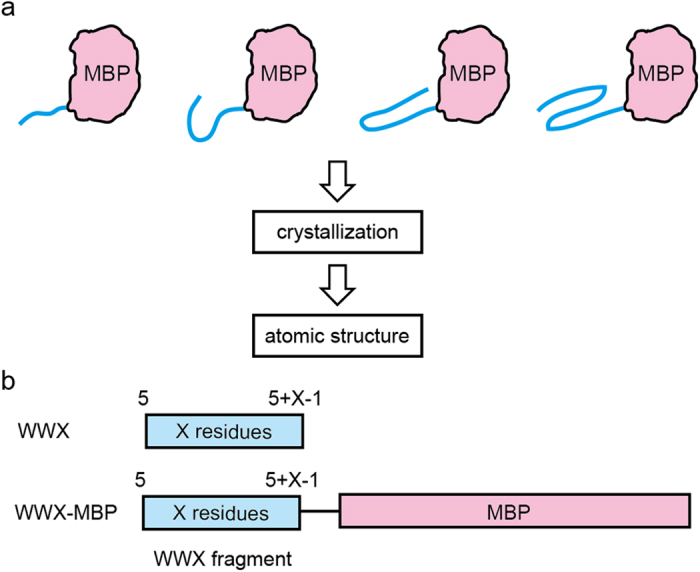
Schematic representation of the models of nascent proteins with increasing amino acid length. (**a**) Our strategy for elucidating the structures of nascent proteins fused with MBP. (**b**) The N-terminal fragments of the WW domain fused with MBP investigated by X-ray crystallography.
