# Supplementary material for: Structural studies of the N-terminal fragments of the WW domain: Insights into co-translational folding of a beta-sheet protein
Source: Sci Rep. 2016 Oct 4;6:34654. doi: 10.1038/srep34654 (PMC5048162; doi:10.1038/srep34654)
Supplement: Supplementary Information [file srep34654-s1.pdf]

Supplementary Information

Structural studies of the N-terminal fragments of the WW domain: Insights into co-translational folding of a beta-sheet protein

Yuya Hanazono, Kazuki Takeda, Kunio Miki*

Department of Chemistry, Graduate School of Science, Kyoto University, Sakyo-ku, Kyoto, 606-8502, Japan

---

Correspondence: Dr. Kunio Miki, Department of Chemistry, Graduate School of Science, Kyoto University, Sakyo-ku, Kyoto 606-8502, Japan

Phone: +81-75-753-4029

Fax: +81-75-753-4032

email: miki@kuchem.kyoto-u.ac.jp

Supplementary Table 1 | Data collection and refinement statistics

	WW11 (Form I)	WW11 (Form II)	WW17	WW19	WW35
**Data collection**					
Space group	$C222_1$	$P4_12_12$	$P2_12_12_1$	$P2_12_12_1$	$P2_1$
Cell dimensions					
a, *b*, *c* (Å)	97.5, 126.1, 173.5	115.6, 115.6, 55.6	51.2, 73.0, 100.8	48.3, 57.7, 124.2	84.5, 120.4, 110.7
α, β, γ (°)	90.0, 90.0, 90.0	90.0, 90.0, 90.0	90.0, 90.0, 90.0	90.0, 90.0, 90.0	90.0, 97.8, 90.0
Resolution (Å)	50-2.40 (2.44-2.40)[a]	50-2.40 (2.44-2.40)	50-2.00 (2.03-2.00)	50-1.90 (1.93-1.90)	50-2.30 (2.34-2.30)
R_{sym}	0.131 (0.558)	0.097 (0.414)	0.050 (0.150)	0.084 (0.386)	0.097 (0.402)
$I / \sigma I$	14.4 (3.7)	29.7 (7.4)	33.2 (11.5)	20.8 (6.0)	10.3 (1.9)
Completeness (%)	99.5 (99.1)	100 (100)	99.8 (99.2)	99.4 (98.1)	95.4 (89.3)
Redundancy	6.3 (6.1)	13.6 (13.1)	7.0 (6.6)	6.4 (6.0)	2.7 (2.1)
**Refinement**					
Resolution (Å)	38.57-2.40	40.86-2.40	29.70-2.00	33.60-1.90	49.01-2.30
No. reflections	41943	15351	26116	27852	92779
R_{work} / R_{free}	0.171/0.214	0.217/0.265	0.154/0.194	0.158/0.198	0.181/0.224
No. atoms					
Protein	5920	2926	3004	2970	12523
Ligand/ion	59	23	23	23	92
Water	591	95	345	402	1117
B-factors (Å^2)					
Protein	24.1	42.6	25.9	19.4	35.4
Maltose	20.6	28.8	16.5	13.3	24.3
Water	31.3	38.4	33.7	28.4	37.3
R.m.s. deviations					
Bond lengths (Å)	0.008	0.004	0.007	0.008	0.004
Bond angles (°)	1.1	0.8	1.1	1.1	0.8

[a] Values in parentheses are for highest-resolution shell.

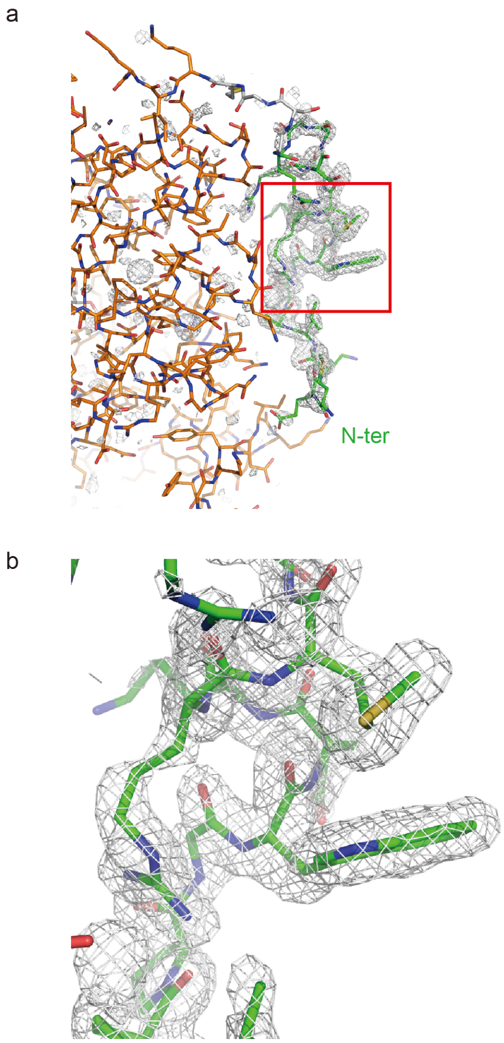

Supplementary Figure 1: Electron density map of WW17-MBP. (a) The *F*o-*F*c omit map for WW17 is represented as a gray mesh contoured at the 2.5σ level. (b) Close-up view of WW17.

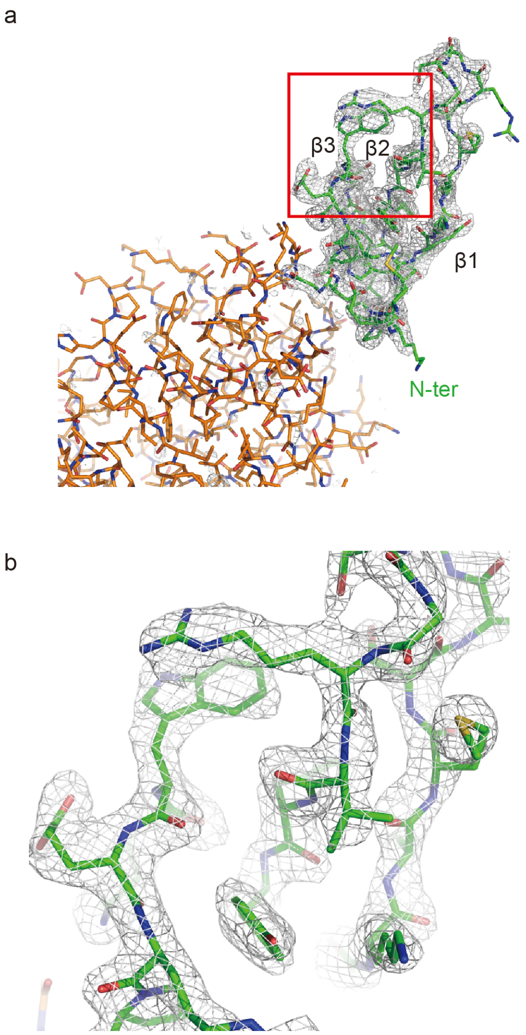

Supplementary Figure 2: Electron density map of WW35-MBP. (a) The *F*o-*F*c omit map for WW35 is represented as a gray mesh contoured at the 2.5σ level. (b) Close-up view of WW35.

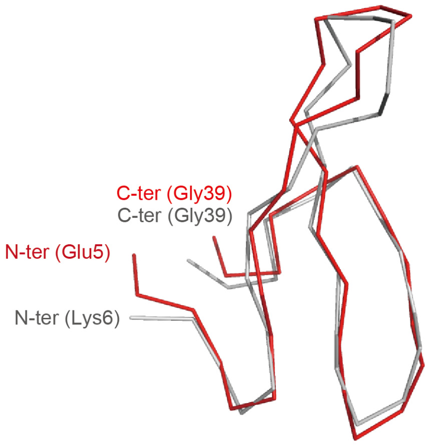

Supplementary Figure 3: Comparison of WW35 (red) and the previously reported structure of the WW domain (PDBID: 1PIN) (gray).

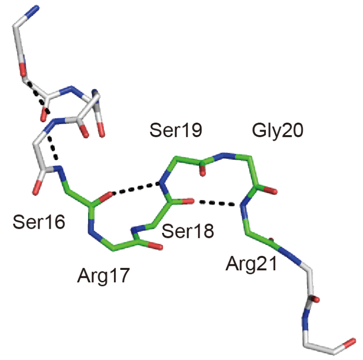

Supplementary Figure 4: Backbone diagram of the loop 1 region (residues Ser16–Arg21) of WW19. Hydrogen bonds are shown as black dashed lines. Residues 16–21 are colored green and the others are colored gray.

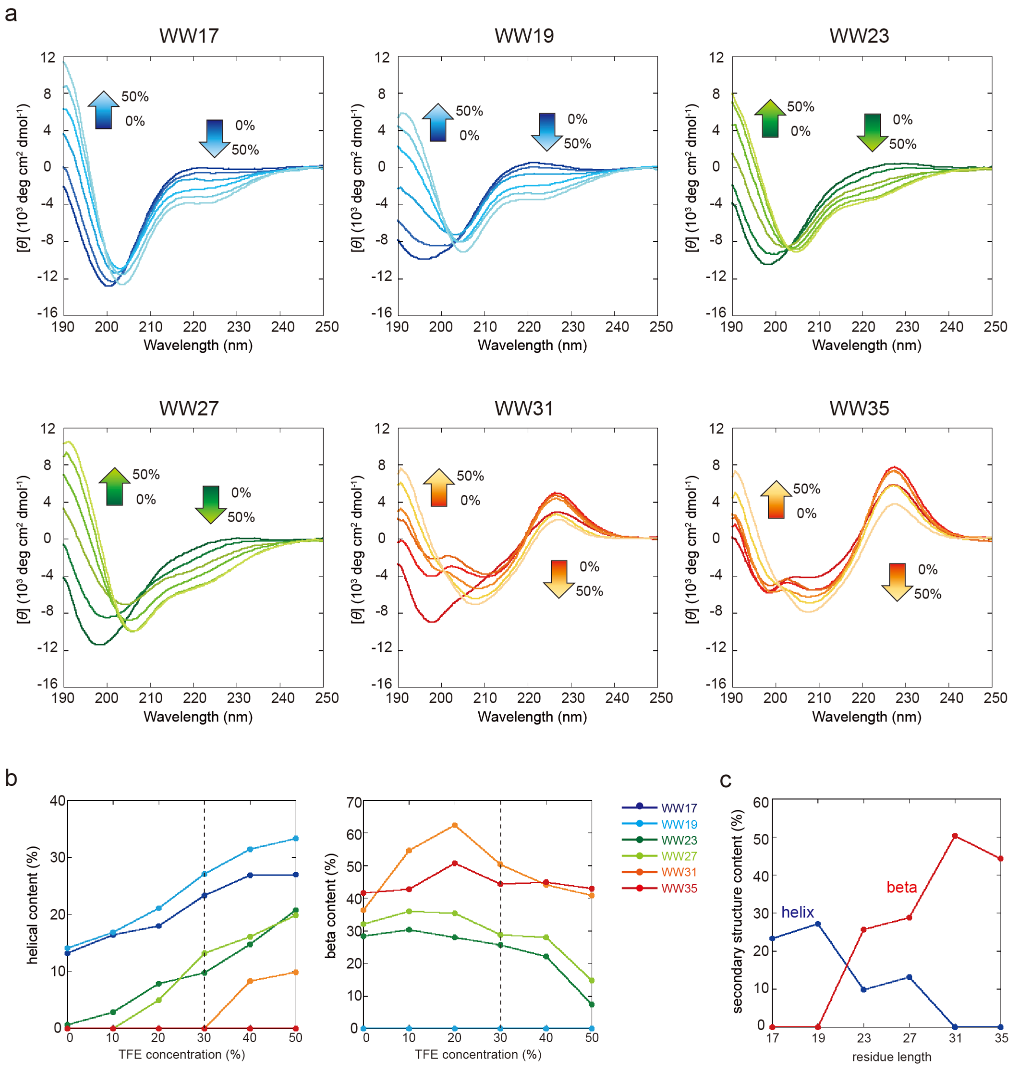

Supplementary Figure 5: CD spectroscopy of the WW domain. (a) CD spectra of the WW domain fragments as a function of the TFE concentration. The gradations of color from dark to light correspond to the TFE concentration gradient from 0% to 50%. (b) Changes of the secondary structure contents as a function of the TFE concentration. WW17 (blue), WW19 (cyan), WW23 (dark green), WW27 (light green), WW31 (orange) and WW35 (red) are shown as solid lines. (c) Secondary structure contents of WW domain fragments under the condition of a 30% TFE concentration. Helical contents and beta contents are shown as red and blue lines, respectively.

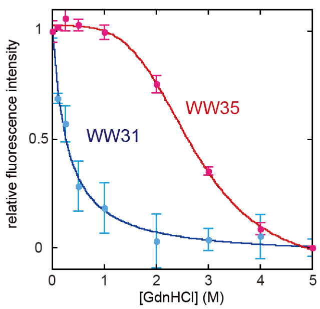

Supplementary Figure 6: Changes in fluorescent emission intensities at 340 nm. The denaturation curves of WW31 (blue) and WW35 (red) are shown as solid lines. Error bars represent standard deviation of the mean for three independent experiments.

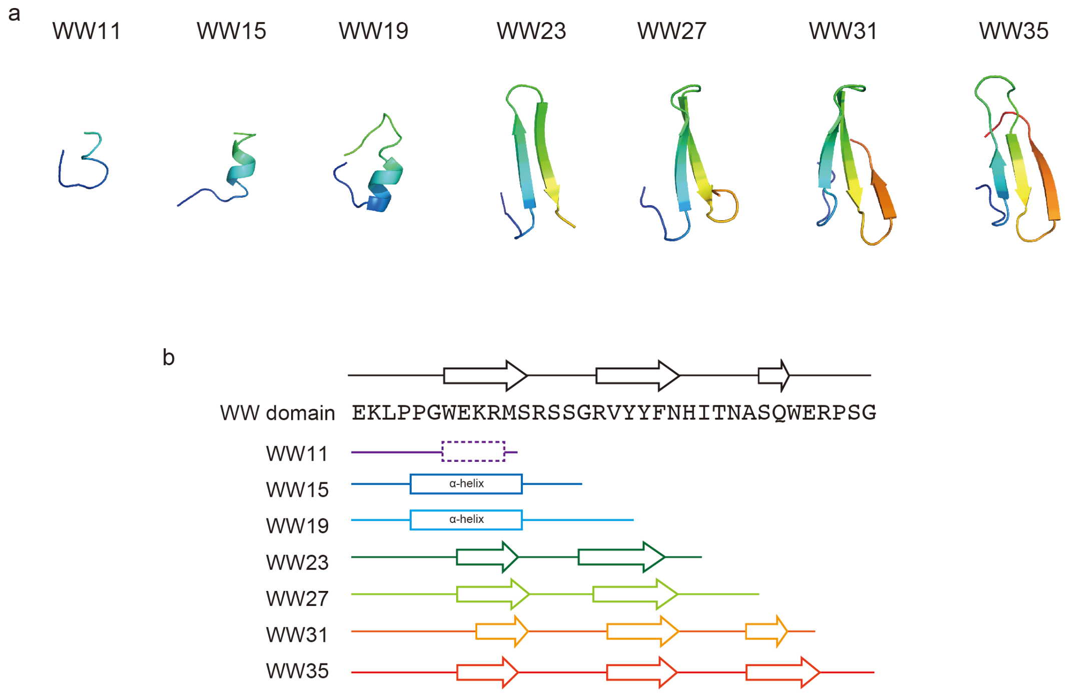

Supplementary Figure 7: Conformational modeling by the PEP-FOLD program. (a) The most representative of the best model for each of WW11, WW15, WW19, WW23, WW27, WW31 and WW35. (b) Secondary structure of the best model. The cylinders and arrows indicate alpha helices and beta strands, respectively. The cylinder formed with a dashed line indicates a helical structure that is not assigned by the program DSSP.

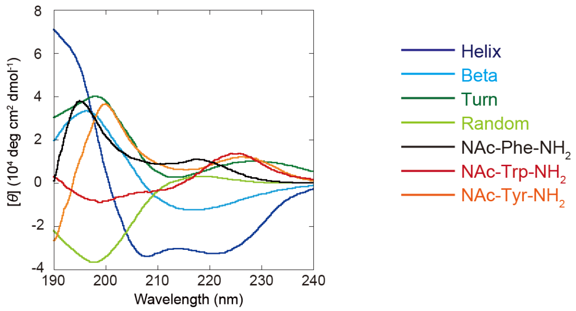

Supplementary Figure 8: Reference data set for secondary structure prediction. The reference data reported by Reed and Reed for helix, beta, turn and random are shown as blue, light blue, green and light green lines, respectively. The spectra of N-acetyl-L-phenylalanineamide, N-acetyl-L-tryptophanamide and N-acetyl-L-tyrosineamide are shown as black, red, and orange lines, respectively.
